# Telling the Stories of Neuroscientific Discovery to Schoolchildren and the Public Can Make an Impact

**DOI:** 10.1523/ENEURO.0078-24.2024

**Published:** 2024-04-04

**Authors:** John A. Pollock

**Affiliations:** Department of Biological Sciences, School of Science & Engineering, Duquesne University, Pittsburgh, Pennsylvania 15282

## Significance Statement

Neuroscience research demands focused attention built upon a foundational knowledge that can encompass the full sweep of science and engineering including, among other disciplines, psychology, biology, chemistry, physics, and computer science. Neuroscience studies range from evolution of life-forms to new innovations in computational modeling. Neuroscientists can look at the population-level behavior, activity of the human brain, or atomic-level resolution of essential molecules. And yet, within these depths of emerging knowledge, the neuroscience community has the capacity to share what we know with young people and the public at large. Even little actions of communicating science in a manner that is broadly accessible and fun can initiate that ripple effect that informs a young mind.

## The Challenge

Television and social media are dominated with advertisements for online shopping, insurance companies, and cell phone plans (Statista, 2022). Furthermore, a number of mental health advertising campaigns are on the rise. Mara Einstein noted that “having companies take an interest in mental health is better than the alternative” (Einstein, 2023). Chris Larson, writing for Behavioral Health Business (Larson, 2022), found that during the Super Bowl and the Olympics, programming with very large audiences, there was a high concentration of ads relating to mental health. The big business of mental health care in America, including self-help apps and video counseling, is becoming more prevalent in the wake of the coronavirus disease (COVID)-19 pandemic. But why is it so prominent? One reason is that consumers now care about mental health differently than in the past, especially among Gen Z (Brown, 2023). Amelia Henderson, writing for the consumer research organization GWI (Henderson, 2023), noted that their “data shows that consumers, particularly younger audiences, generally like [mental health awareness] campaigns: 73% of Gen Z support mental health messaging in ads.”

There is a deeper reason for all this advertising. Mental health challenges are rampant for all levels of the American population, from kids to seniors. Furthermore, it is becoming less stigmatizing to talk about one's mental health status. Consider that years ago, it was taboo to talk about one's cancer diagnosis. Cancer disease has largely lost its stigma because we now know that you cannot “catch” cancer from being near a cancer patient. It is also the case that there are many powerful therapies to treat cancer. Similarly, and however slowly, the message that mental health **is** health is beginning to find inroads into the national conversation. It seems that Gen Z (those born between the mid-1990s and early 2010s) are a motivating force for this new national conversation. As a generation, they grew up having a different collection of social traumatic experiences that have been amplified by social media: from school shootings, 911, political vitriol, the economic hardship of 2008/2009, and COVID-19. Yet even though the conversation is started, the social media advertisements and apps or services still leave a lot out of what mental health really is.

Mental health is now being recognized as a global issue. The World Health Organization reported in 2022 that as many as one in eight people live with a mental disorder (WHO, 2022), and they recently released the *Mental Health Gap Action Programme* guidelines for improving the access to high-quality evidence-based and meaningful mental health care in low- and middle-income countries (Brohan et al., 2024). The country of South Africa, for example, apparently has an opportunity to take an even bolder step with the publication of a National Mental Health Policy Framework and Strategic Plan 2023–2030 and a proposed National Health Insurance Plan for emphasizing the goal of holistic health care (Shisana et al., 2024). With the pervasive incidence of mental health challenges throughout the world, and the emerging interest for change in governmental policies, there is an important opportunity for the neuroscience community to participate.

The basis of mental health has its roots in the fundamental evolution and biology of the nervous system. The visceral feelings, emotions, and thoughts that align with mental health are a product of our vertebrate physiology. To survive, all mammals need to maintain basic bodily functions, like body temperature, hydration, oxygen tension, and blood pH. Under normal circumstances, metabolic processes keep these bodily systems in a very specific functional range. This is homeostasis, which involves a web of feedback sensory mechanisms for normal physiology. When the stress response is triggered, homeostasis is disrupted with rapid changes in the animals' physiology to deal with the stressor. The changes that occur during a stressful period are known as allostasis (McEwen, 2017). The autonomic nervous system and the hypothalamic–pituitary–adrenal (HPA) axis are two of the major physiological systems that respond to stress in mammals. However, most people don't know anything about homeostasis and allostasis. And yet, by understanding the aspects of one's own physiology, by knowing what's going on, an individual has more agency and is empowered to exert personal control. Control might be as simple and immediate as a mindfulness moment, a little more sleep, or being kind to our gut microbiome. That is where outreach and informal science, technology, engineering, and math (STEM) education can play a role and where neuroscientists can find ways to make an impact, large or small.

## The Approach

Historically, informal science education is played out in self-selecting tours of museums or the choice to watch informative broadcast television like *Watch Mr. Wizard*, with Don Herbert creating a remarkable 547 episodes from 1951 to 1965 (LaFollette, 2002). With *Mr. Wizard* revived in the 1970s and again in the 1980s, the show went on to inspire the likes of *Bill Nye the Science Guy* (Rockman, 1996, 2007*)*, *Beakman's World* (Jensen, 2006), and even *MythBusters* (Schwarts, 2006), among others. Yet they all had a format that is not that much different than a classroom lecture. While clearly effective, there are other ways to introduce science that can have a different level of engagement.

The Partnership in Education (partnershipineducation.com) at Duquesne University creates analog and digital learning tools for students 10 years old and above. Kids learn through fun with games and stories that can be used in the classroom, in after-school activities, or at home. Several of the projects are focused on neuroscience topics which relate to neurobiology that kids experience. By engaging the students from the perspective of their own world view, the anticipated outcome is that these kids will see that they can understand science and that it is okay to ask questions and expect answers about how the world works. In a fun and natural way, the scientific method is put on display not through a lecture but through the kids’ own experience or the experience of someone “just like them” who is portrayed in a story. Furthermore, if kids have these positive experiences with science before adolescence hits, we anticipate that it is more likely that they will grow with an appreciation for scientific discovery and carry that innate curiosity into adulthood. Creating opportunities to see science in action is not going to make every student grow up to be a neuroscientist, engineer, or physician. It is, however, more likely that they will grow into adulthood knowing what science can tell them about their everyday lives.

The Partnership in Education has a focus on mental health. Adolescence is a dynamic process of growth and change across biological domains as well as cognitive, social, and sense of self-esteem. It is a time during a young person's life where emotions, problems, and everyday life can seem overwhelming. Several studies (Bitsko et al., 2022; Gramlich, 2023) note that there is a growing epidemic of mental health challenges among today's youth that encompasses depressive disorders, stress and anxiety, withdrawal, loneliness, and low self-esteem, among others. While there are multiple defined and undefined pressures that are influencing this problem, we believe that there are interventions and learning opportunities that can help students develop an understanding of their physiological processes that influence mental health and that they can use this information to build stress management skills and resiliency for life. This is important because we know that the consequences of unmitigated stress can be poor performance in almost all aspects of adolescent life: school and grades, sports and the arts, self-image, and relationships. The impacts of stress can also include self-abusive behaviors such as use of alcohol or drugs, self-harm, and even the disease of suicide (Czeisler et al., 2020). An array of surveys and reports (Bitsko et al., 2022; Jones et al., 2022; Gramlich, 2023) reveal that 37% of high school students currently report mental health challenges and over 75% of parents worry about their kids struggling with anxiety or depression. There is a critical need to address these mental health challenges.

While most state and federal teaching standards for K–12 do not explicitly include mental health topics, we advocate that mental health **is** health. Currently, in many situations, aspects of mental health are treated as secondary to physical health. Furthermore, mental health has a biological basis and, as such, can be translated into accessible science and health topics for ready introduction into the classroom. Our approach is to start addressing the concepts and reality of stress and anxiety earlier in a student’s academic life through biology or health lessons. Starting in the early preteen years of late elementary grades, and providing resources that extend to early high school, an integrated collection of digital and analog multimedia products supported with supplementary classroom curriculum provides students with knowledge of their own biology, as well as strategies, skills, and techniques for managing common mental health challenges. Using a backward design methodology (Wiggins & McTighe, 2005; McTighe & Wiggins, 2012; Kantorski et al., 2019), students' self-identified needs and preferences are put at the forefront of the design process. Backward design methodology first identifies what is to be learned and then how to assess learning and finally to plan classroom instruction.

## Addressing Mental Health as Health

It is relevant to work from the point of view that a direct and explicit approach for addressing mental health makes the most sense. The use of overt messaging that is authentic and genuine will help to destigmatize issues of mental health. This approach, applied in an age-appropriate manner, empowers students with skills for managing “every day” stressors and helps to establish mental strength and resiliency. With the right skills and self-awareness, the students can resolve issues before they snowball, or importantly, they can recognize that an issue at hand is of big enough concern that adult or professional help is needed.

As a counterpoint, in many situations, mental health is treated as secondary to physical health. For example, if a student shows signs of a fever, cough, and stomachache, whether at home or at school, parents and teachers alike seek to help the student and treat their illness right away with home remedies or a visit to the pediatrician; the aim is to head off more severe symptoms. On the contrary, it is rare that minor symptoms of disruptive mental health are similarly treated when symptoms are first identified. If we can begin treating mental health like physical health, with simple preventative self-management behaviors, a broadened awareness of symptoms, and an open willingness to seek help when things are not right, then students will carry appropriate skills forward into young adulthood so that they can address other life challenges.

## Creative Interventions

### Infographics

**Figure 1.**
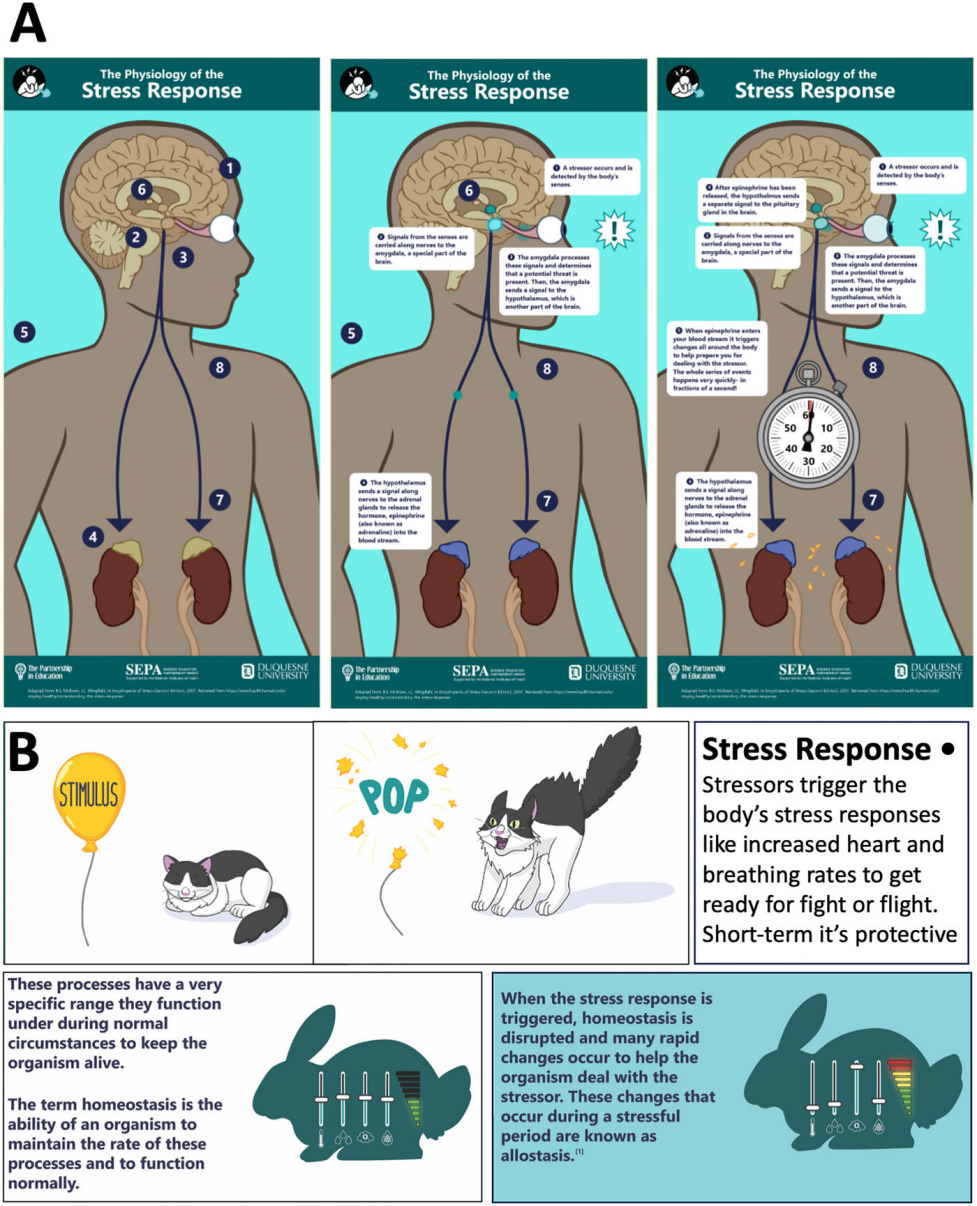
InterFACTives are infographic pages that illustrate aspects of the biology of stress and anxiety. Simple graphics with concise text help to reveal aspects of the fundamental biology and neuroscience of stress and anxiety. ***A*** presents the HPA axis. ***B*** highlights aspects of the stress response and, separately, homeostasis and allostasis. (Google Chrome thepartnershipineducation.com/resources/all-about-stress).

Creating a simple infographic web resource that maps out key aspects of stress and anxiety can provide students with access to knowledge. The Partnership in Education produced a series of researched, concise web pages that address the following: (1) *What is Stress?* (NIH), (2) *Physiology of the Stress Response* (McEwen, 2007), (3) *Fight or Flight* (Gleitman, 2004; Schmidt et al., 2008), (4) *Homeostasis and Allostasis* (McEwen, 2017), (5) *Stress in the Wild and at Home* (Romero, 2020), (6) *Long Term Effects of Stress* (Lloyd et al., 2005; Kang et al., 2012; Mariotti, 2015), as well as (7) *What is Anxiety?* (Goldin et al., 2009; Duval et al., 2015). Each web page (also available as a pdf) provides a brief fun and accessible explanation that teachers can use in capsule lessons of discovery. Crafted from the basis of science, storyboarded, and vetted by other experts, the web-based interactive graphics let the user explore, for example, the physiology of stress with an overview of the HPA axis, highlighting the role of the amygdala, the hypothalamus, adrenal glands, and the pituitary gland with epinephrine and cortisol (Fig. 1*A*). Other infographics reveal that animals and pets can experience stress, too (Fig. 1*B*).

### Narrative

**Figure 2.**
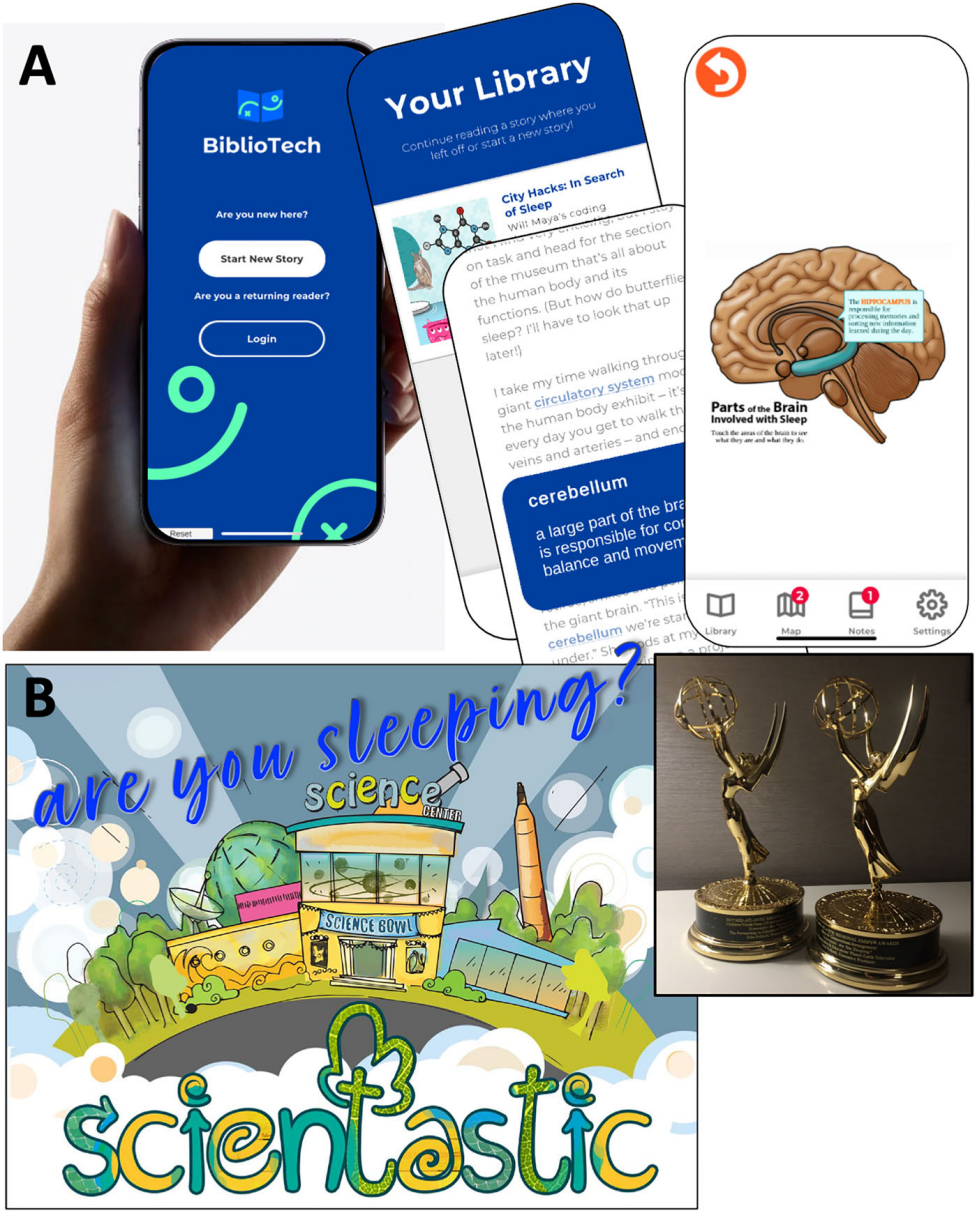
Digital media for understanding the importance of sleep. ***A***, The BiblioTech *CityHacks: In Search of Sleep* is a user-friendly iOS/Android app with branched build-your-own-adventure story, games, a digital notebook, interactive graphics, and videos. The Adaptive Reader feature allows users to adjust the reading level. ***B***, *Scientastic! Are You sleeping?* is a two-time Emmy Award-winning television program focused on the importance of sleep, complementing the companion BiblioTech Adaptive Reader *CityHacks: In Search of Sleep* (Kantorski et al., 2019).

Another approach is achieved with a longer-format narrative, broadcast television, and a story book in a digital platform. In one scenario, we address the notion that adequate sleep sets the stage for several aspects of brain health and mood management. To give students learning tools that address the importance and functions associated with sleep, we used the iPhone operating system (iOS)/Android BiblioTech format (Kantorski et al., 2019; Kantorski et al., 2020). The story *CityHacks: In Search of Sleep* (Kantorski et al., 2019) was developed to complement the Emmy Award-winning television show Scientastic! Are you Sleeping? (Pollock et al., 2014; Fig. 2). The story arcs in *Scientastic! Are You Sleeping* and of BiblioTech *CityHacks: In Search of Sleep* were created by the Partnership in Education based on the science and biology of sleep. Then, professional writers were engaged to craft the scripted stories, with attention to scientific detail and accuracy.

An additional BiblioTech story, *Rebound: Beating Concussions*, was similarly created. In this case, the aim was to address the increasing number of sports-related concussions in children (Elbin et al., 2016). In consultation with Drs. Michael Collins and Anthony Kontos and colleagues at the University of Pittsburgh Medical Center Concussion and Sports Medicine Center (Broglio et al., 2015; Collins et al., 2014; Elbin et al., 2016; Henry et al., 2016; Kontos et al., 2016), the Partnership in Education again created a story arc, which was then developed into a full narrative. *Rebound* also introduced a new interactive feature that allows users to adjust the complexity of the story text, changing the reading level on the fly. Termed the Adaptive Reader (Kantorski et al., 2020), it allows users to adjust the text complexity of the story to better match their individual reading abilities. The adjustment occurs in the vocabulary and sentence structure, while the narrative, STEM content, and learning goals remain the same.

### Evaluation

These stories were all created through an iterative design process that engaged students and their teachers in identifying relevant storylines and characters that they wanted to see. Additional feedback was collected on early scripts, and prototypes before the finalized versions were produced. Pre-/postsurvey instruments were used to assess content knowledge gains, shifts in attitudes, as well as students' overall enjoyment (Kantorski et al., 2019; Kantorski et al., 2020). The assessment of *CityHacks* demonstrated that after using the app, fifth-grade students' self-reported knowledge about the science of sleep was significantly greater than before they used the app for concepts of sleep deprivation, rapid eye movement sleep, circadian rhythm, and biphasic sleep patterns. Similarly, their self-reported knowledge about neuroscience was significantly greater after using the app (Kantorski et al., 2019). Furthermore, after a single use of the app, students were significantly more likely to be able to identify factors that can affect one's quality of sleep from before (*M* = 17%) to after (*M* = 61%); *t*_(17)_ = −3.688; *p* = 0.002. They were also significantly better at identifying the benefits of a later school start time for teenagers from before (*M* = 11%) to after (*M* = 33%); *t*_(17)_ = −2.204; *p* = 0.042 (Kantorski et al., 2019).

The assessment of *Rebound: Beating Concussions* focused on middle school and early high school student athletes, as well as parents and coaches (Kantorski et al., 2020). After using the app, the data showed that the study participants had knowledge of significantly more concussion symptoms and significantly more concussion treatments including brain rest, physical therapy, and even nutrition (Kantorski et al., 2020). The study also found that the *Rebound* story increases knowledge and understanding about concussions among student athletes, parents, and coaches. The results of this study indicate that the BiblioTech format can effectively engage diverse audiences and provide critically needed concussion-related education.

The innovation of the Adaptive Reader technology is valuable. In the simplest terms, with the Adaptive Reader, the narrative can be presented in three formats: a narrative written at fourth-, fifth-, and seventh-grade reading levels. Assessed with Flesch–Kincaid grade-level readability scoring and other techniques (Kantorski et al., 2019), we recognize that Flesch–Kincaid did not necessarily represent the whole picture. As such, we consider that there are two factors in text difficulty and leveling: (1) sentence length and word complexity and (2) semantic content and vocabulary. Nearly all leveling tools—Flesch–Kincaid, Gunning fog, simple measure of Gobbledygook, etc.—only measure text complexity. Using a combined approach that acknowledges the limitations of Flesch–Kincaid, we compared and edited the different reading levels of the text using Lexile and Dale–Chall tools along with the Educational Development Laboratory *Core Vocabulary* book. Ultimately, we analyzed the text section by section for story consistency across the reading levels of the text. The result is an important advantage that with the Adaptive Reader, all students can participate in class discussions and feel confident about their ability to understand the relevant STEM concepts with independence from feeling that the text is too hard or too easy. We believe that the Adaptive Reader is uniquely empowering.

### Short-format animation

**Figure 3.**
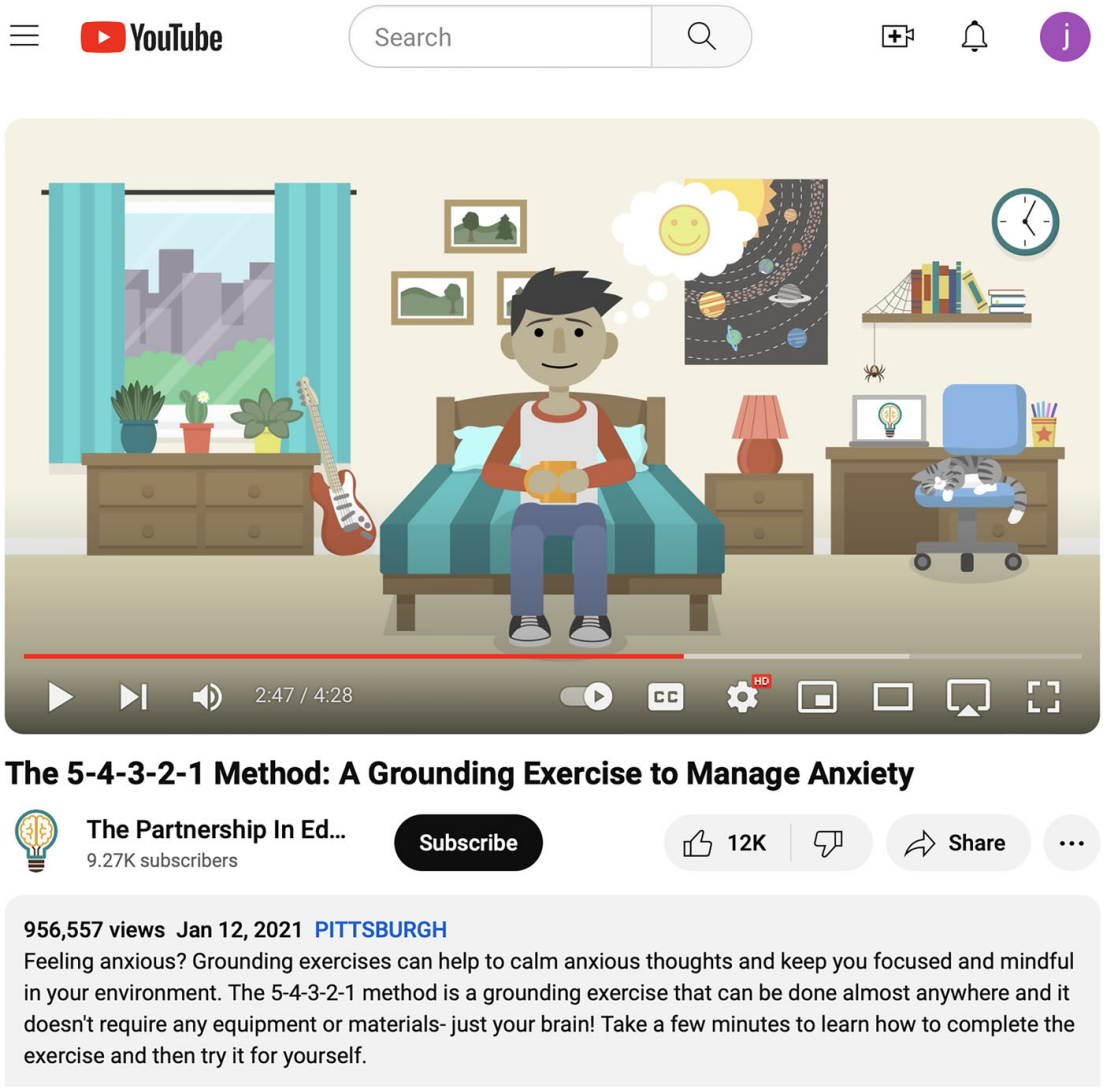
YouTube video *5-4-3-2-1 Method: A Grounding Exercise*. One of the five current YouTube videos of @ThePartnershipinEducation addressing different mindfulness practices.

We are producing short, animated videos that are hosted on the YouTube channel @ThePartnershipInEducation, which has nearly 10,000 subscribers and has accrued ∼1.4 million views, with an additional ∼55,000 views occurring every month. The most popular videos are from a collection that covers topics of mindfulness and meditation. Each of these mindfulness videos present simple, low-barrier techniques for controlled calming and relaxation. Topics include 5 min yoga, a 90 s equal breathing exercise, and a guided movement meditation as well as one entitled *The 5-4-3-2-1 Method: A Grounding Exercise* (Fig. 3). This mindfulness exercise teaches viewers how to calm anxious thoughts and keep one's focus grounded in their immediate environment. In <5 min, the viewer can learn how to complete the exercise and then try it for themselves. It can be done almost anywhere, and it does not require any equipment or materials—just one's brain!

Each video is created through a development pipeline that starts with concept development, a review of relevant scientific literature, scripting, storyboarding, and user feedback. Critical review edits the script, while concept art is refined, all prior to producing the animation with freelance voice-over talent to create a complementing narrative with an appropriate and accessible voice that is tailored to the target audience.

## Conclusion

As a neuroscientist, separately studying the fundamental biology of pain and pain relief (Saleem et al., 2019; Deal et al., 2022, 2023), I find that there is opportunity to develop different types of learning resources that can have an important impact on the community at large. Across the neuroscience community, projects both large and small can play an essential role. By working from one's strengths and by engaging college students and colleagues from across campus to help, collaborations and partnerships can succeed. Telling the stories of science and putting them in a context that is relevant to the world view of young people improves learning far beyond memorization of facts. With the burgeoning social media opportunities of digital platforms, a simple message can reach a wide audience. Of paramount importance is well founded, researched, and scientifically supported messages that, when made relatable, invite people into fostering their identity as someone who can understand science and that they belong in a STEM community.

As a scientific community, messages that we communicate are an extension of what E. L Boyer (Boyer, 1990; Boyer et al., 2015) challenged us to recognize that we have an obligation for our scholarship to extend beyond the research of the lab and classroom, to reach across campus to other academic cultures (Snow, 1959) and through the gates to include public engagement.

## Synthesis

Reviewing Editor: Christophe Bernard, INSERM & Institut de Neurosciences des Systèmes Decisions are customarily a result of the Reviewing Editor and the peer reviewers coming together and discussing their recommendations until a consensus is reached. When revisions are invited, a fact-based synthesis statement explaining their decision and outlining what is needed to prepare a revision will be listed below. The following reviewer(s) agreed to reveal their identity: NONE.

The paper is nice as is. Some typos to correct.
